# PI3Kα in cardioprotection: Cytoskeleton, late Na^+^ current, and mechanism of arrhythmias

**DOI:** 10.1080/19336950.2019.1697127

**Published:** 2019-12-02

**Authors:** Pavel Zhabyeyev, Xueyi Chen, Bart Vanhaesebroeck, Gavin Y. Oudit

**Affiliations:** a Department of Medicine, University of Alberta, Edmonton, Canada; b Mazankowski Alberta Heart Institute, University of Alberta, Edmonton, Canada; c UCL Cancer Institute, London, UK

**Keywords:** PI3Kα, cytoskeleton, late sodium current, arrhythmias, heart failure

## Abstract

PI 3-kinase α (PI3Kα) is a lipid kinase that converts phosphatidylinositol-4,5-bisphosphate (PIP2) to phosphatidylinositol-3,4,5-triphosphate (PIP3). PI3Kα regulates a variety of cellular processes such as nutrient sensing, cell cycle, migration, and others. Heightened activity of PI3Kα in many types of cancer made it a prime oncology drug target, but also raises concerns of possible adverse effects on the heart. Indeed, recent advances in preclinical models demonstrate an important role of PI3Kα in the control of cytoskeletal integrity, Na^+^ channel activity, cardioprotection, and prevention of arrhythmias.

## Introduction

Phosphoinositide 3-kinases (PI3Ks) phosphorylate phosphatidylinositol lipids in intracellular membranes at the 3ʹ position of the inositol ring. Class I PI3Ks act at the plasma membrane by phosphorylating phosphatidylinositol-4,5-bisphosphate (PIP2) to produce phosphatidylinositol-3,4,5-triphosphate (PIP3). In addition to catalytic functions, class I PI3Ks also act as scaffold proteins to create regulatory complexes independent of the kinase action of PI3Ks [1–3]. Class I PI3Ks have four isoforms (PI3Kα, PI3Kα, PI3Kγ, and PI3Kδ) consisting of a p110 catalytic and regulatory subunit. Based on the regulatory subunit preference, class I PI3Ks are grouped into class IA enzymes (p110β, p110β, and p110δ), which bind to a p85 family regulatory subunit, and the class IB PI3K (p110γ), which binds to p84 or p101 regulatory subunit. PIP3 produced by these PI3Ks binds with effectors that have a PI3K-lipid-binding pleckstrin homology (PH) domain. These effector proteins, including the AKT Ser/Thr protein kinase, regulate various biological processes such as nutrient sensing, survival, cell cycle, migration, and others [3–8]. Different isoforms are activated by distinct mechanisms: PI3Kα and PI3Kδ are activated by receptor tyrosine kinase (RTK) and Ras small GTPases, PI3Kγ is activated by Gαγ subunits released following G protein-coupled receptor (GPCR) activation and by Ras, and PI3Kα can be activated by RTKs, Gαγ and the Cdc42 and Rac small GTPases. The catalytic subunits, p110β, p110β, p110γ, and p110δ, are encoded by the *PIK3CA, PIK3CB, PIK3CG*, and *PIK3CD* genes, respectively. Upregulation of class IA PI3K signaling is frequently found in cancer and occurs through various mechanisms, including inactivation of the PI3K antagonizer phosphatase and tensin homolog (PTEN), overactivation of RTKs upstream of PI3K and gain-of-function somatic mutations in genes coding for catalytic subunits [9–11]. Among PI3K gene mutations, mutations in *PIK3CA* are the most frequent, with much lower frequency in *PIK3CB* and *PIK3CD* [12]. The crucial role of the PI3K pathway in cancer development and progression made this pathway a promising target for cancer treatment [13–15]. However, the development of PI3K-targeted drugs has raised a need to investigate the role of PI3K isoforms in wider physiology and pathophysiology. Recent preclinical studies have revealed that PI3Ks plays a critical role in hypertrophy, electrical remodeling, cardiovascular diseases, including cytoskeletal regulation during heart failure, cardioprotection from ischemic injury, and channel activity regulation [6–8,16,17]. In this review, we will focus on the novel role of PI3Kα as a modulator of cytoskeletal integrity, channel activity, Ca^2+^ cycling, and the mechanisms underlying arrhythmogenicity upon PI3Kα inhibition.

## PI3K inhibitors in cancer therapy

The involvement of various PI3K isoforms in cancer made them a prime target for cancer therapies [13–15]. The PI3Kα isoform is the main target for solid tumors, and PI3Kδ is targeted in hematological tumors, whereas PI3Kα and PI3Kγ receiving less attention (Table 1). Since PI3Kα is the functionally-dominant isoform expressed in the heart, in this review, we will focus on the cardiac effects of PI3Kα inhibition.

**Table 1.** PI3K isoform-specific and pan-PI3K inhibitors.

Agent	Target	Phase	Cancer type/Condition	Major Toxicities	Cardiac specific	Notes	Ref
Alpelisib	PI3Kα	I	Advanced breast cancer	Hyperglycemia, dermatologic adverse effects, gastrointestinal discomforts, fatigue	QTc prolongation		[18–20]
		I	Advanced solid tumors				[21,22]
		I	Advanced colorectal cancer		QTc prolongation		[23]
		I	Advanced ovarian, fallopian tube, primary peritoneal, or breast cancer				[24]
		II	Early Breast cancer			No additional benefits with letrozole	[25]
serabelisib		I	Advanced solid tumors	As above, with in addition elevated AST/ALT			[26]
Taselisib		I	Advanced solid tumors	Gastrointestinal discomforts, fatigue, hyperglycemia, dermatologic adverse effects, stomatitis, colitis		Target mutant PI3Kα isoform > wild-type PI3Kα, δ, γ > PI3Kβ	[27]
		I	Advanced solid tumors, HR-positive advanced breast cancer				[28]
		II	Advanced HER2-negative, HR-positive breast cancer				[29]
		Now approved	Estrogen receptor-positive, *PIK3CA*-mutant, locally advanced or metastatic breast cancer				[30]
GSK2636771	PI3Kβ	I	Advanced solid tumors	Gastrointestinal discomforts, fatigue, hypophosphatemia, hypocalcemia			[73]
IPI-549	PI3Kγ	I	Advanced solid tumors	Gastrointestinal discomforts, dermatologic adverse effects, pyrexia, elevated AST/ALT			[74]
Umbralisib	PI3Kδ	I	Relapsed or refractory chronic lymphocytic leukemia or lymphoma	Gastrointestinal discomforts, fatigue, dermatologic adverse effects, hypokalemia, hematological toxicities (neutropenia, anemia, thrombocytopenia), elevated AST/ALT, pneumonia, colitis		Inhibits casein kinase 1ϵ as well	[75]
		I	Relapsed or refractory chronic lymphocytic leukemia or mantle cell lymphoma				[76]
Idelalisib		I, II	Relapsed indolent non-Hodgkin lymphoma (NHL)	fatigue, gastrointestinal discomforts, dermatologic adverse effects, pyrexia, hematological toxicities (neutropenia, anemia, thrombocytopenia), elevated AST/ALT, pneumonia, colitis		Combined with lenalidomide and rituximab: hepatoxicity and immune suppression[77]	[78–81]
		I, III	Relapsed or refractory CLL		Hypokalemia		[82–85]
		II	Chronic lymphocytic leukemia or small lymphocytic leukemia			treatment-naive older patients; AEs-related death: pneumonitis, sepsis	[86,87]
		II	Relapsed or refractory CLL or NHL			terminated early due to pneumonitis in 18% of patients; 2 AE-related death: pneumonitis	[88]
		II	Relapsed or refractory Hodgkin lymphoma			60% deaths during the study or long term follow up (1 death occurred on study – hypoxia)	[89]
		–	B-cell prolymphocytic leukemia			Case study n = 5	[90]
		I	Relapsed mantle cell lymphoma and relapsed follicular lymphoma			Idelalisib with lenalidomide and rituximab; terminated due to serious toxicity;	[91]
		I	Allergic rhinitis	Nasopharyngitis, myalgia			[92]
Nemiralisib		II	Persistent, uncontrolled asthma	Cough		Administered through inhaler; negative results	[93]
Copanlisib	Class I pan- PI3Ks	I	Advanced solid tumors and non-Hodgkin’s lymphomas	Hyperglycemia, gastrointestinal discomforts, hypertension, dermatologic adverse effects, fatigue, mucositis, elevated aspartate aminotransferase and alanine aminotransferase, thrombocytopenia, neutropenia, anemia, pneumonitis	Hypertension		[94]
		I	Advanced or refractory solid tumors				[95]
		I	Advanced cancer		As above with atrial fibrillation, sinus tachycardia		[96]
		II	Indolent or aggressive malignant lymphoma	As above with pancreatitis, infection	As above with cardiac disorders (not being specified in the article)		[97]
Buparlisib (BKM120)		I	Advanced solid tumors	Gastrointestinal discomforts, hyperglycemia, fatigue, dermatologic adverse effects, stomatitis, elevated transaminase, hypertension, psychiatric disorders, confusion, increased lipase, increased serum amylase	Hypertension		[98]
		Ib	HER2-positive advance or metastatic breast cancer				[99]
		I	Metastatic renal cell carcinoma			7 in 28 patients discontinued therapy because of toxicity	[100]
		I, III	Hormone receptor-positive metastatic breast cancer				[101–103]
		I	Relapsed/refractory acute leukemias				[104]
		II	Advanced or recurrent endometrial carcinoma			Stopped before end of recruitment for toxicity	[105]
		II	Recurrent glioblastoma				[106]
		I	Metastatic breast cancer	As above with neutropenia, peripheral neuropathy			[107]
		II/III	Advanced or metastatic breast cancer				[108]
		I	High-grade ovarian and breast cancer	As above with thrombocytopenia, leukopenia, anemia, lymphopenia			[109]
		I	Advanced solid tumors				[110]
		Ib	Advanced solid tumors	As above with increased creatine kinase			[111]

AST/ALT, the ratio of aspartate transaminase (AST) to alanine transaminase (ALT); CCL, chronic lymphocytic leukemia; HR, hormone receptor; NHL, non-Hodgkin lymphoma

Clinical trials of inhibitors that block PI3Kα commonly reported hyperglycemia as the major side effect [18–30], which is unsurprising considering the critical involvement of PI3Kα in insulin signaling [31,32]. Corrected QT (QTc) prolongation was observed for alpelisib (BYL719) [18,23], but not for serabelisib (MLN1117) [26] or taselisib (GDC0032) [27] (Table 1). General inhibition of PI3K and/or tyrosine kinase activity had been linked to cardiotoxicity and drug-related heart failure [13,14]. Pan-PI3K inhibitors exhibit similar cardiac side effects as PI3Kα inhibition suggesting that the effects might be due to inhibition of this PI3K. So far, arrhythmogenic side effects are known for the pan-PI3K inhibitor copanlisib [15]. For copanlisib, which now has regulatory approval, prolonged QT_c_ (ΔQT_cB_ ≥ 60 ms) was found in 6.6% of patients, prompting a request by the FDA for further monitoring [15].

Tyrosine kinase inhibitors may indirectly suppress PI3Kα. Inhibition of PI3Kα has been put forward as an explanation of the arrhythmogenic effects of ibrutinib [33]. Only ibrutinib (Bruton tyrosine kinase inhibitor) has been linked to instances of atrial fibrillation, ventricular arrhythmias, and sudden cardiac death [34–36].

## Cardiac effects of PI3Kα inhibition in diabetes

In murine models of diabetes, reduced sensitivity to insulin is associated with diminished PI3Kα activity which has been linked to both hyperglycemia and arrhythmias [37,38]. Prolongation of the action potential and QTc interval was observed in different animal models of diabetes [39,40]. The reduced PI3Kα activity causes the dis-inhibition (activation) of late Na^+^ current leading to prolongation of the action potential [37,38]. Conversely, upregulation of PI3Kα activity in the heart has been shown to protect from ventricular arrhythmias and sudden death associated with pathological hypertrophy and heart failure [17,41].

## PI3Kα in cardioprotection

PI3Kα signaling has recently emerged as an important cardioprotective pathway. In murine animal models, PI3Ka pathway has been shown to be protective in the model of tamoxifen toxicity [42] and various models of heart failure [6,43,44]. For pressure overload model of heart failure, a recent study by Patel et al. [6]. elucidated a mechanism underlying the accelerated progression of heart failure observed in a murine model of PI3Kα deficiency, suggesting that PI3Kα activation is part of a compensatory response during heart failure. They also reported reduced PI3Kα activity in human and dog hearts with dilated cardiomyopathy, additionally suggesting that PI3Kα is a part of compensatory response mechanisms to maintain heart function under adverse conditions [6]. In the murine model of ischemic preconditioning, PI3Kα was also found to be the key PI3K isoform to limit myocardial infarct size [43]. In the murine model of doxorubicin-induced heart failure, the loss of PI3Kα exacerbates cardiac atrophy, leading to biventricular atrophy associated with right ventricular dysfunction [44]. Similarly, patients receiving anthracyclines and trastuzumab, which indirectly inhibits PI3Kα activity, exhibit biventricular dysfunction and reduced heart mass [45]. Taken together, the PI3Kα pathway appears to play a crucial cardioprotective role.

## PI3Kα in compensatory response during heart failure

**Figure 1.**
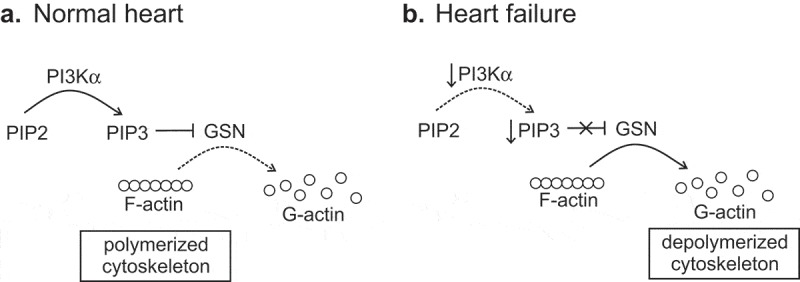
Regulation of actin cytoskeletal integrity by PI3Kα in the normal heart and heart failure. (a) Normal heart: PI3Kα produces PIP3, which inhibits gelsolin (GSN) activity preventing actin severing action of GSN and favoring a polymerized state of the cytoskeleton (prevalence of F-actin). (b) Heart failure: diminished PI3Kα activity results in reduced PIP3 levels, which leads to active GSN severing F-actin and depolymerized cytoskeleton (prevalence of G-actin).

Under quiescent conditions, lack or reduced PI3Kα activity does not significantly affect heart function [6,46,47], but lack of PI3Kα activity is known to accelerate heart failure progression in the pressure overload model of heart failure [6,47]. However, the exact mechanisms of this increased susceptibility to heart failure were unknown. Recently, Patel et al [6]. proposed that in response to biomechanical stress, PI3Kα is recruited to intercalated disks and the plasma membrane where it produces PIP3, which is required to suppress the activity of gelsolin (GSN), an actin-severing protein. When PI3Kα activity is suppressed, GSN activity is markedly increased, leading to lower levels of actin polymerization and a less resilient actin cytoskeleton. Tissue of human and dog hearts with dilated cardiomyopathy also showed reduced levels of actin polymerization, and in human samples, there was a negative correlation between cardiac function and actin depolymerization (the lower ejection fraction corresponded to higher depolymerization levels) [6]. In addition, human and canine hearts with dilated cardiomyopathy showed reduced PI3Kα activity. In a murine dilated cardiomyopathy model, the exacerbation of cardiac dysfunction in PI3Kα-deficient mice was prevented by experimental GSN deficiency, suggesting that PI3Kα is an important *in vivo* cytoskeletal regulator during cardiac remodeling in pressure overload heart failure. In the proposed framework [6], PI3Kα produces PIP3 which suppresses GSN activity, preventing depolymerization of the actin cytoskeleton by GSN (Figure 1a). In the case of heart failure, reduced PI3Kα activity leads to low PIP3 levels and increased GSN activity, which in turn favors the depolymerization of the actin cytoskeleton (Figure 1b). Another possible mechanism of cardioprotection mediated by PI3Kα is suppression of late Na^+^ current by PI3Kα-generated PIP3 [7,48]. Since activation of late Na^+^ current accompanied heart failure in the pressure overload model[49], lack of PI3Kα activity and the ensuing reduction in PIP3 to suppress late Na^+^ current may contribute to the accelerated transition to heart failure. The link between PI3Kα inhibition, late Na^+^ current, Ca^2+^ cycling, and arrhythmias is discussed in more detail below.

## PI3Kα and QT prolongation

*PI3Kα upregulation and QT*. PI3Kα activity controls expression levels of many channel forming proteins (K^+^: Kir, Kv, TASK; Ca^2+^: Cav1; Na^+^: SCN5A). In murine models, an increase in PI3Kα activity, for example, due to exercise leads to an increase in the protein levels of K^+^, Ca^2+^, and Na^+^ channels as well as their current densities [17]. Increasing PI3Kα activity via expression of constitutively-active PI3Kα also produces higher protein levels and current densities [41]. Overall, upregulation of PI3Kα due to exercise or overexpression did not affect QT interval due to balanced increase in protein levles of both repolarizing K^+^ channels (Kir, Kv, TASK) and depolarizing channels (Ca^2+^: Cav1; Na^+^: SCN5A). Moreover, PI3Kα upregulation was protective against arrhythmias, pathological hypertrophy, and dilated cardiomyopathy [16,17,41].

*PI3Kα inhibition prolongs QT*. Over the last decade, there has been a steady accumulation of observations linking pharmacological inhibition of PI3Kα to activation of late Na current (I_Na-L_). Apparently, some classical blockers of rapidly-activating delayed rectifier K^+^-channels, such as dofetilide and E4031, can also inhibit PI3Kα activity and activate I_Na-L_ [50]. In patients, ibrutinib (inhibitor of Bruton tyrosine kinase, an upstream effector of PI3Kα) increased cardiac disorders (2-fold) and atrial fibrillation (3-fold) [34], as well as instances of sudden death and ventricular arrhythmias [35,36]. In mice, high doses of ibrutinib produce analogous results (an increase in susceptibility to induced atrial and ventricular arrhythmias) and was associated with inhibition of PI3Kα activity [51].

**Figure 2.**
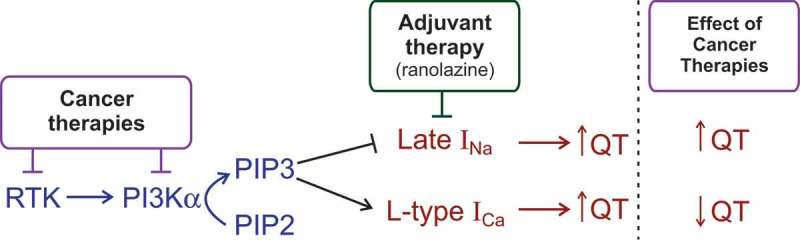
Cancer therapies prolong QT interval via inhibition of PI3Kα. Inhibition of PI3Kα activity either at receptor tyrosine kinase (RTK) step or directly at Pi3Kα will lead to a reduction in PIP3 levels, which exert an inhibitory effect on late I_Na_. In the absence of PIP3-related inhibition, additional depolarizing I_Na_ will prolong action potential and QT interval. The QT prolongation could be moderated in large mammals due to the opposite effect of PIP3 on L-type Ca^2+^ current (I_Ca_). Reduction in PIP3 levels will translate in the smaller amplitude of depolarizing current I_Ca_, which will favor QT shortening.

In murine models, inhibition of PI3Kα produced QT prolongation or long-QT (LQT) and was associated with activation of I_Na-L_ [50], whereas in canine cardiac myocytes the use of pan PI3K inhibitors lead to inhibition of delayed rectifier K^+^ currents and activation of I_Na-L_ [52]. The isoform-specific PI3Kα inhibitor (BYL719) increased I_Na-L_ and resulted in a triggered activity in murine cardiomyocytes [48] and isolated murine hearts [7,53], but had no effect on murine K^+^ currents [7]. These results suggest a straightforward link between PI3Kα activity, the prolongation of the action potential, and QT interval (Figure 2). In this framework, an indirect inhibition of PI3Kα activity by cancer therapies by receptor tyrosine kinase-based therapies (e.g., ibrutinib) [34–36] or directly (e.g., alpelisib) [18,23] may reduce PI3Kα activity leading to reduced PIP3. Since PIP3 suppresses I_Na-L_, a reduction in PIP3 levels will dis-inhibit (activate) I_Na-L_, which as a depolarizing current will promote action potential and result in QT prolongation (Figure 2). This QT prolongation due to PI3Kα inhibition may be somewhat compensated in large mammals (including humans) by the influence of PIP3 on L-type Ca^2+^ current (I_Ca,L_). Indeed, PIP3 has *stimulatory* effects on depolarizing L-type Ca^2+^ current (I_Ca,L_); therefore, the reduction of PIP3 levels due to PI3Kα inhibition will promote QT prolongation *via* I_Na-L_ and counteract it *via* I_Ca,L_ (Figure 2). A promising approach therefore to prevent QT prolongation is to block the activation of I_Na-L_ with adjuvant therapy (*e.g*., ranolazine) (Figure 2) [7]. Besides direct pro-arrhythmic effects of I_Na-L_ activation, the increased I_Na-L_ may potentially contribute to the development dilated cardiomyopathy since increased influx of Na^+^ due to gain-of-function mutations in *SCN5A* and *SCN10* (genes encoding Na^+^ channels) has been implicated in the development of heart failure in rodents [49] and was associated with dilated cardiomyopathy [54] as well as sudden cardiac death [55,56]. Another implication of increased I_Na-L_ activity is sarcoplasmic reticulum Ca^2+^ overload, which we will discuss below.

## PI3Kα, Ca^2+^ cycling, and triggered arrhythmias

**Figure 3.**
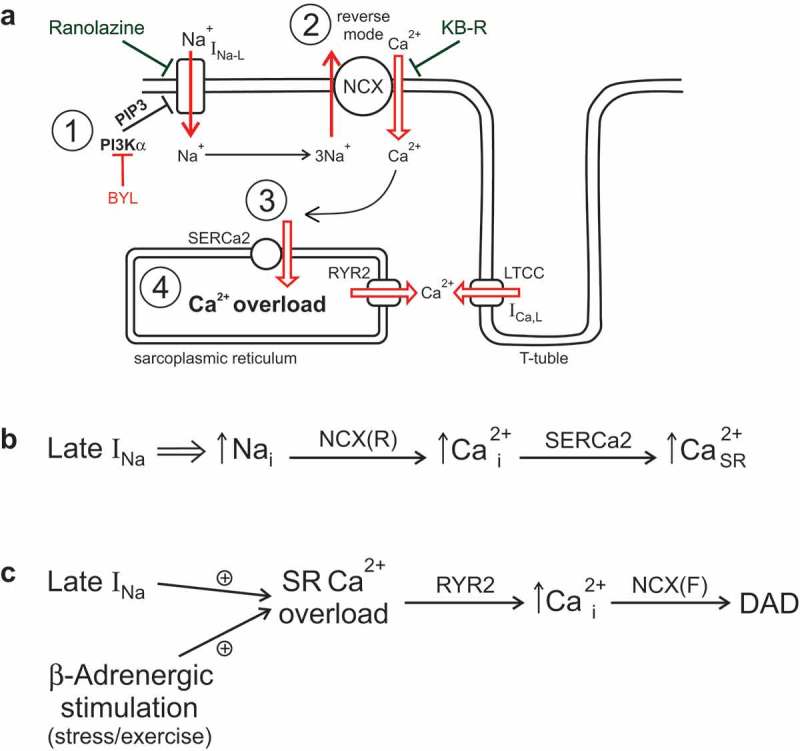
Effect of PI3Kα on Ca^2+^ cycling, α-adrenergic stimulation, and arrhythmias. (a) Effect of the PI3Kα inhibition on Ca^2+^ cycling. Inhibition of PI3Kα (1) reduces the inhibitory action of PIP3 on late Na^+^ current (I_Na-L_). Increased I_Na-L_ will generate an influx of Na^+^, which will promote the influx of Ca^2+^ via Na^+^-Ca^2+^ exchanger (NCX) (2). Increased Ca^2+^ influx and thus increased cytosolic Ca^2+^ will stimulate additional Ca^2+^ uptake via sarco-endoplasmic reticulum Ca^2+^ ATPase type 2 (SERCa2) (3) leading to increased Ca^2+^ levels in sarcoplasmic reticulum or Ca^2+^ overload (4). (b) Schematic representation of the sequence of the events depicted in *A*. (c) Interaction of activation of late I_Na_ and α-adrenergic stimulation. Both late I_Na_ and α-adrenergic stimulation are known to contribute to sarcoplasmic (SR) Ca^2+^ overload. The SR Ca^2+^ overload may result in spontaneous Ca^2+^ release (increase in cytoplasmic Ca^2+^_i_) via ryanodine receptor channels (RYR2). An increase in cytoplasmic Ca^2+^ will produce depolarizing current via the forward mode of NCX (NCX(F)) leading to arrhythmogenic delayed afterdepolarization (DAD).

Dis-inhibition of I_Na-L_ due to inhibition of PI3Kα [7,48,50,52] can exacerbate Ca^2+^ overload by modulating Ca^2+^ cycling and α-adrenergic stimulation [7], both of which are important contributors to the development of several arrhythmias [56–58]. In this framework, dis-inhibited I_Na-L_ will produce an additional Na^+^ influx (I_Na-L_; see (1) in Figure 3a), which will increase cytosolic Ca^2+^ either *via* a reverse mode of Na^+^-Ca^2+^ exchanger at the plateau of action potential (2) or by reduction of Ca^2+^ extrusion *via* forward mode during resting potential. Increase in cytosolic Ca^2+^ will facilitate Ca^2+^ uptake to the sarcoplasmic reticulum (SR) *via* SERCa2 (3) leading to Ca^2+^ overload (4) (Figure 3a,b) [7]. This Ca^2+^ overload will promote prolongation of the action potential, abnormal automaticity, early and delayed afterdepolarization, and increased dispersion of repolarization [59,60]. This increase in SR Ca^2+^ load is additive to α-adrenergic stimulation [7] and thus will create a risky situation similar to catecholaminergic polymorphic ventricular tachycardia (CPVT) [58,61]. The combined effect of I_Na-L_ and α-adrenergic stimulation will lead to an excessive Ca^2+^ load that may result in spontaneous Ca^2+^ release, which will generate depolarizing current (I_NCX_) *via* forward mode of NCX producing delayed afterdepolarization (DAD) and possibly triggered activity (premature action potential) (Figure 3c) [7]. In this framework, excessive Ca^2+^ overload can be prevented either by inhibition of I_Na-L_ by ranolazine or reverse mode of NCX by KB-R7943 (Figure 3a) [7].

## PI3Kα inhibition and heart failure in the clinic

Besides the arrhythmogenic effects of PI3Kα inhibition associated with I_Na-L_ activation and related Ca^2+^ overload, these processes may contribute to the development and exacerbate heart failure. The activation of I_Na-L_ and increased Ca^2+^ influx *via* NCX have been linked to the development of heart failure in a murine pressure overload model [49] *via* hypertrophic calcineurin-NFAT signaling [62]. In heart failure, when α-adrenergic signaling is upregulated to maintain cardiac output [63], an additional Ca^2+^ from I_Na-L_-NCX axis would compound with the effects of α-adrenergic stimulation resulting in the accelerated progression of heart failure. Pro-arrhythmic effects of PI3Kα inhibition will be amplified because of the higher levels of Na^+^-Ca^2+^ exchanger protein observed both in human failing heart [64] and in rodent models of heart failure [65].

This means that the risk of cardiac-specific side effects of PI3Kα inhibition will be greater in the elderly patients who are more likely to suffer from heart failure or preexisting cardiac dysfunctions [66]. Polymorphisms in genes involved in all steps that produce Ca^2+^ overload (Figure 3a,b) could also contribute to susceptibility of PI3Kα-dependent cardiac side effects. Polymorphisms and mutations in *SCN5A* and *SCN10A* (genes that are responsible for Na^+^ influx *via* I_Na-L_) have already been linked to dilated cardiomyopathy, arrhythmias, and sudden cardiac death [54–56,67]. Other LQT-related polymorphisms and mutations may aggravate QT prolongation due to PI3Kα inhibition exacerbating arrhythmic risk. Additionally, since PI3Kα inhibition leads to Ca^2+^ overload, polymorphisms and mutations related to CPVT, especially the ones that increase sensitivity to Ca^2+^ overload [58], will also magnify arrhythmogenic effects PI3Kα inhibition. The link between genetic background and arrhythmogenic effects of PI3Kα inhibition warrants further in-depth studies.

Currently, there are two possible approaches to mitigate cardiotoxicity related to PI3Kα inhibition. One is the use of an I_Na-L_ blocker (e.g., ranolazine) that will prevent AP prolongation and Ca^2+^ overload resulting from inhibition of PI3Kα [7,68]. Ranolazine is known to improve heart function in heart failure patients (not related to drug-induced cardiotoxicity) [69–71] as well as to prevent anthracycline-induced cardiotoxicity [72]. The other less explored approach is to block the reverse mode of NCX; however, currently, there are no approved drugs to achieve this effect.
